# Relationships between egg-recognition and egg-ejection in a grasp-ejector species

**DOI:** 10.1371/journal.pone.0166283

**Published:** 2017-02-07

**Authors:** Manuel Soler, Francisco Ruiz-Raya, Gianluca Roncalli, Juan Diego Ibáñez-Álamo

**Affiliations:** 1 Departamento de Zoología, Facultad de Ciencias, Universidad de Granada, Granada, Spain; 2 Behavioral and Physiological Ecology group, Centre for Ecological and Evolutionary Studies, University of Groningen, Groningen, The Netherlands; 3 Dept. Wetland Ecology, Estación Biológica de Doñana, C.S.I.C, Avda. Sevilla, Spain; University of Akron, UNITED STATES

## Abstract

Brood parasitism frequently leads to a total loss of host fitness, which selects for the evolution of defensive traits in host species. Experimental studies have demonstrated that recognition and rejection of the parasite egg is the most common and efficient defence used by host species. Egg-recognition experiments have advanced our knowledge of the evolutionary and coevolutionary implications of egg recognition and rejection. However, our understanding of the proximate mechanisms underlying both processes remains poor. Egg rejection is a complex behavioural process consisting of three stages: egg recognition, the decision whether or not to reject the putative parasitic egg and the act of ejection itself. We have used the blackbird (*Turdus merula*) as a model species to explore the relationship between egg recognition and the act of egg ejection. We have manipulated the two main characteristics of parasitic eggs affecting egg ejection in this grasp-ejector species: the degree of colour mimicry (mimetic and non-mimetic, which mainly affects the egg-recognition stage of the egg-rejection process) and egg size (small, medium and large, which affects the decision to eject), while maintaining a control group of non-parasitized nests. The behaviour of the female when confronted with an experimental egg was filmed using a video camera. Our results show that egg touching is an indication of egg recognition and demonstrate that blackbirds recognized (i.e., touched) non-mimetic experimental eggs significantly more than mimetic eggs. However, twenty per cent of the experimental eggs were touched but not subsequently ejected, which confirms that egg recognition does not necessarily mean egg ejection and that accepting parasitic eggs, at least sometimes, is the consequence of acceptance decisions. Regarding proximate mechanisms, our results show that the delay in egg ejection is not only due to recognition problems as usually suggested, given that experimental eggs are not touched significantly more often. Thus, the delay in egg ejection is mainly the consequence of a delay in the decision to eject, probably triggered by mechanical constraints imposed by eggs that are harder to eject (i.e. larger). Our results offer important information on the relationships between recognition and ejection and contribute to a better understanding of host defences against brood parasites.

## Introduction

Avian brood parasites impose strong selection for evolution of defensive traits on their hosts because brood parasitism frequently leads to a total loss of host fitness. This is the case in the common cuckoo (*Cuculus canorus*, hereafter “cuckoo”) given that the cuckoo chick usually ejects all host offspring from the nest [1]. Therefore, many hosts have evolved defences, which can function at any stage of the breeding cycle (pre-laying, laying, nestling and fledging; [2]). The most common and efficient defence used by hosts is recognition and ejection of the parasitic egg [3–5]. Many experimental egg-recognition studies have led to major advances in the study of co-evolution [6–12].

Egg recognition experiments are the most efficient tool used in the study of the relationships between brood parasites and their hosts. Pioneer naturalists began to study egg discrimination by hosts by introducing alien eggs into their nests as early as the beginning of the nineteenth century [13]. In the 1970s the first properly designed experiments (Rothstein 1970, in [13]) provided the foundation for future studies [7, 8, 10, 14–18]. Numerous studies involving egg recognition experiments have been performed since then (see Appendix in [2]). Aided by technological discoveries, these have enormously advanced our knowledge on host responses to brood parasitism. These advances have elucidated not only the evolutionary and coevolutionary implications of egg recognition and rejection, but also the mechanisms involved in the evolution of this ability that many host species display.

Most studies of egg-recognition behaviour have only dealt with the act of rejection [19]. However, egg rejection is a complex behavioural process [20, 21] consisting of at least three stages: first, the host has to recognize the parasitic egg; second, it has to decide whether or not to reject the putative parasitic egg, and third, the act of ejection itself must take place [21]. Very little is yet known regarding the cognitive processes leading to egg rejection, although the use of cameras in egg-recognition experiments at the beginning of the present century [22] has provided key information that has expanded our knowledge of the proximate mechanisms responsible for egg recognition and rejection decisions [19, 21–25].

The timing of egg ejection is highly variable both among and within host species. Some individuals may eject a foreign egg the same day that it is introduced into the nest (even immediately, within minutes or even seconds), while others delay their response for several days [22, 24, 26–30]. Information on rejection latencies could provide important insights for understanding the proximate mechanisms responsible for egg recognition and rejection decisions. For instance, delayed ejection of the parasitic egg can be provoked by perceptual problems affecting the recognition and decision making processes [24, 30]. On the other hand, delayed rejection is not always caused by recognition problems. Although several studies have provided evidence demonstrating that recognition precedes rejection [19, 22–24], in certain circumstances hosts may decide not to eject the parasitic egg, even if it has been recognized [21, 25, 31]. Recently, it has been shown that the acceptance of parasitic eggs is not exclusively linked to recognition errors, but egg rejection can also be interrupted at later stages of the process such as during the ejection process itself [32].

The blackbird (*Turdus merula*) has been frequently used as a model species in egg-recognition experiments [32–38]. We have detailed knowledge of the response of blackbirds to experimentally introduced parasitic eggs, but not regarding the proximate mechanisms and cognitive processes associated with egg recognition and rejection decisions. For instance, the ejection latency, the interval before egg ejection, has previously been studied in this species (2 and 2.5 days for non-mimetic and mimetic eggs, respectively), showing that the degree of mimicry did not have a significant effect on the timing of rejection decisions [35]. Recently, Ruiz-Raya *et al*. [32] showed in this same species that a trait that does not affect egg recognition (egg mass) hampered the ejection of the parasitic egg suggesting that the act of ejection may be limited by mechanical constraints. Thus, an understanding how different egg traits can influence the host response, including the latency of that response, by affecting different stages of the rejection process is essential given that these may have important implications for the evolutionary relationships between brood parasites and their hosts.

Here we expand previous findings on the acceptance of parasitic eggs (see above) by performing an experimental study using blackbirds as the model species. The blackbird is a grasp-ejector, using the beak to hold and remove eggs. We manipulated two characteristics of the parasitic egg that can affect specific components of the egg rejection process: (a) the degree of colour mimicry, related mainly to the first (recognition) stage; and (b) egg size, which should affect mainly the last stage (the act) of the egg rejection process in grasp ejectors (such as blackbirds, [8, 22, 38]). We video-recorded female behaviour in both experimental and control nests in order to quantify different parameters of the female response to experimentally introduced eggs.

We predicted that: (1) the latency to ejection will be longer for larger eggs because, even if they are recognized, egg volume should be critical for grasp ejectors, given that they have to pick the egg up with their beak. This prediction contrasts with the situation in puncture-ejector species that pierce eggs to hold them, in which the time to ejection is mainly determined by shell thickness rather than egg volume [24, 30]. (2) Latency to ejection will be longer for mimetic eggs in comparison with non-mimetic eggs. Usually, non-mimetic eggs are ejected sooner than mimetic ones [24, 28, 39–41, but see 35], probably because of the greater recognition difficulties involving mimetic eggs [24, 42]. (3a) Experimentally introduced eggs that are recognized should be ejected. This prediction is based on two pieces of evidence. First, grasp-ejection is a nearly cost free method of rejection [8, 38]; and second, consistency in egg-rejection behaviour in blackbirds is very high [35]. Alternatively, (3b) some experimental eggs that are recognized will be accepted. This prediction is based on the the recent demonstration that blackbirds sometimes recognize experimental eggs but do not eject them [32]. For this last prediction, we assumed that egg touching can be considered a good proxy of egg recognition (see Material and Methods). Pecking or “touching” of foreign eggs is a frequent behaviour shown by hosts when confronted with an experimental egg, and it has been considered to indicate recognition, even if ejection does not occur [21–25, 32].

## Material and methods

### Ethics statement

Research has been conducted according to relevant Spanish national (Real Decreto 1201/2005, de 10 de Octubre) and regional guidelines. All necessary permits were obtained from the “Consejería de Medio Ambiente y Ordenación del Territorio de la Junta de Andalucía”, Spain. Approval for this study was not required according to Spanish law since it is not a laboratory study in which experimental animals have to be surgically manipulated and/or euthanized. Our study area is unprotected private land, whose owners allowed us to work on their properties. This study did not involve endangered or protected species.

### Experimental design and data collection

We conducted this study in the Lecrín valley (Southern Spain 36°56'N, 3°33' W). The study area is dominated by orange groves. See [43] for a detailed description of the blackbird population.

We actively searched for blackbird nests in the study area throughout the breeding season from early March to the end of June 2012. Once a nest was located, we checked it to determine its contents. If the nest was found at the building stage we visited it every three days until the first egg appeared.

Our experiment consisted of introducing an experimental egg into nests during the laying stage (after at least two eggs were laid) or during the incubation period. We chose to use both stages because several studies indicate that breeding stage does not affect egg rejection rate in this species [8, 28, 34, 35].

We created six different treatments by combining two features in the model eggs, which allowed us to affect two stages of the egg-rejection process directly (see predictions). In particular, we manipulated (a) the degree of mimicry in terms of colour and (b) the egg size (see Fig 1 in [38]). We used natural fresh eggs painted to be mimetic or non-mimetic and of three different sizes relative to blackbird egg size: (i) small: house sparrow (*Passer domesticus*) eggs, (ii) medium: blackbird eggs and (iii) large: common quail (*Coturnix coturnix*) eggs. Sparrow eggs are similar in size to cuckoo eggs [44], whereas common quail eggs are considerably bigger [38] and allowed us to examine the ejection behaviour of blackbirds when confronted with eggs that were very difficult to grasp-eject, a situation similar to that encountered by small-sized host species. See [32] for detailed information on the masses of the three egg types. We created a seventh group (control) for which we followed the same experimental procedure as for the others (visit frequency, clutch manipulation, filming procedure, etc.; see below) except that no egg was introduced into the nests. Each nest was assigned randomly to one of these seven groups. More detailed information regarding the experimental design, egg painting and the egg models used can be found in [38].

We placed a Panasonic HDC-SD40 video camera near the nest (1.5–2.5 m) to film female activity at the nest for the two hours immediately following the introduction of the experimental egg. During the egg stage, blackbird behaviour is not influenced by placing a camera near the nest [32, 38, 45]. We successfully filmed the behaviour of blackbird females in a total of 106 nests.

After the two recording hours, we checked for the presence of the introduced egg in the nest in order to determine the “immediate ejection rate”. If the model egg was still in the nest after the first two recording hours, we checked again after 24 hours and daily for the following five days to determine ejection latency: the interval until ejection, and the long-term ejection rate or egg acceptance. The results for the long-term ejection rate have been previously published [38] and are not included in the Results section of this paper; however, they have been used in the “Time to ejection” and “Relationships between recognition and ejection” subsections, topics that were not studied in the earlier paper [38]. We considered the experimental egg to have been accepted when it remained in the nest for five days. If it disappeared during this five-day interval (see [38] for more details about the choice of this five-day period) while the remaining eggs were intact and warm, we assumed the parents had ejected the egg, finishing the trial. In such cases, we assigned an ejection interval considering that ejection had occurred between the last two visits, adding 12 hours to the time (in hours) of the last visit in which the introduced egg was still present. Eggs from deserted nests were collected and kept in a refrigerator at 5°C for use on subsequent days. Each experimental egg was only used once and then discarded.

The recordings were viewed using a 3.5 Plus KM Player. Each recording was carefully examined to extract information on the following variables for each nest: (1) first-contact touches during the first visit (the number of times the female touches the eggs before settling on the nest during her first return to the nest during the experimental period), (2) first-contact touches per visit (similar to variable 1 but taking all visits together and corrected by the number of visits), (3) incubation touches (the number of times the incubating female touches the eggs corrected by the time spent in the nest), and (4) the egg inspection time for all visits combined.

### Relationships between egg touches and egg recognition

Weak pecking or touching of foreign eggs by hosts is probably a tactile method that allows birds to gather information about the identity of an egg (own or foreign), its state of incubation and/or the potential costs of ejecting it [21]. Blackbird females “touch” experimental eggs so weakly (see S1 Video) that it is very difficult to distinguish this behaviour from touches performed to move their own eggs during incubation, unless the beak is clearly visible when touching the eggs. A recent study has found that sometimes eggs in control nests are also “touched” [32]. For this reason we have called this behaviour in this species egg-touching instead of egg-pecking. However, we consider that egg-touching is also an indication of recognition in blackbirds because egg-touching is much more frequent in experimental than in control nests [32], as also found in the present study, (2.57 ± 0.45 touches per visit in experimental nests; 0.71 ± 0.23 touches per visit in control nests). Also, another blackbird study found that the number of touches in a nest did not vary after the introduction of a conspecific (i.e. highly mimetic) egg, whereas they increased significantly following the introduction of a non-mimetic egg [46]. Thus, to study the relationships between recognition and ejection we have assumed that egg-touching indicates recognition and, to be conservative, we considered an experimental egg to have been recognized (even if it was not ejected) when it was touched more frequently than in 95% of control nests (i.e. percentile 95).

### Statistical analyses

We used generalized linear models (GLM) in order to test the effect of our treatment, i.e. the interaction between degree of colour mimicry and egg size, on immediate ejection rate (binomial error and logit link function) and the interval to ejection (Gamma error and log link function). We built our maximal model by including the following predictors: egg colour, egg size, clutch size, their interactions and date. During model simplification, non-significant terms were dropped and models were fitted by using different link functions. Akaike’s Information Criterion (AIC) was used to evaluate the resulting models. Following Zuur *et al*. [47], we performed Zero-affected negative binomial models (ZANB or *hurdle*) by using *pscl* (R package v.1.4.9 [48]) in order to cope with zero-inflation and overdispersion in our egg recognition variables. For these variables, the significance of effects was assessed from likelihood ratio tests (LRT) for nested models by using *lmtest* (R package v.0.9–34 [49]). Differences in first-contact touches between the first and last visits were assessed by means of generalized linear mixed models (GLMM) including female identity as the random factor while considering zero-inflation. For that, we used the *glmmADMB* package (v. 0.8.3.3 [50]) and then Wald tests were generated by using the *car* package [51]. *Post-hoc* analyses of interactions were performed by using the *phia* package (v. 0.2–1 [52]). We did not include large eggs in the analysis of ejection latency since there was no variation in their response (only one large non-mimetic egg was ejected). We also performed correlation analyses in order to identify some possible relationships between ejection latency and three different variables: inspection time, first-contact touches on first visit and first-contact touches per visit. Values provided are means ± SE. All analyses were performed using R version 3.2.3 [53]. Data used in this paper are included in S1 Table.

## Results

### Immediate ejection

We managed to film female behaviour when confronted with an experimental egg in 85 experimental and 21 control blackbird nests. In 13 of the filmed nests the experimental egg was ejected, always by the female, during the two hours of filming (immediate ejection). Colour mimicry had a significant effect on the immediate ejection rate (χ^2^ = 14.84, df = 1; *p* < 0.001). Thus, non-mimetic experimental eggs were ejected significantly more often during the two first hours than mimetic experimental eggs. Size also had a significant effect on immediate ejection (χ^2^ = 7.37; df = 2; *p* = 0.03) as small experimental eggs were ejected significantly more often than large eggs (z = 2.15, *p* = 0.03). No differences were found between medium and large (z = 1.28, *p* = 0.20) or between medium and small experimental eggs (z = 1.24, *p* = 0.22). Clutch size, date and all interactions between predictors did not significantly affect the immediate ejection rate (all p-values > 0.1).

### Ejection latency

**Fig 1:**
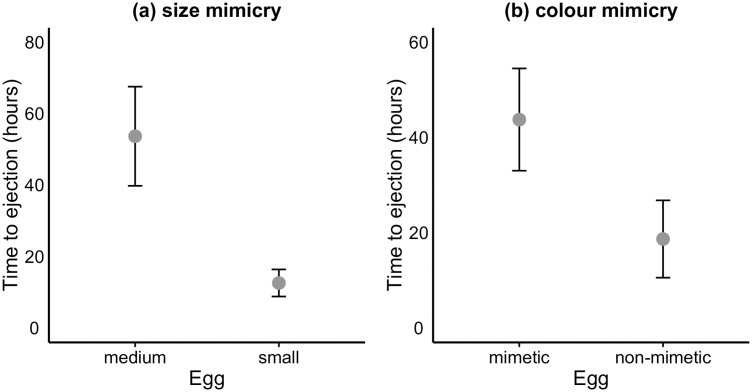
Time needed by for female blackbirds to eject an introduced egg with respect to (a) size mimicry and (b) colour mimicry.

The size of model eggs affected the ejection latency (F_1, 27_ = 12.9, *p* = 0.001). Small model eggs were ejected sooner than medium-sized eggs (12.62 ± 3.79 h and 53.66 ± 13.87 h, respectively; Fig 1a), which is in agreement with Prediction 1. Furthermore, we detected a significant effect of colour on ejection interval (F_1, 26_ = 8.9, *p* = 0.006), non-mimetic eggs being ejected sooner (18.69 ± 8.11 h) than mimetic eggs (43.74 ± 10.73 h; Fig 1b), which supports Prediction 2. This effect of colour on ejection latency is also supported by the fact that 63.2% of the ejections of non-mimetic eggs, but only of 9.1% of mimetic eggs, occurred within the first two hours after their introduction, i.e. during the filming period. However, we did not find any effect of the interaction between colour and size (F_1, 26_ = 1.06, *p* = 0.31).

Finally, it is worth noting that the ejection latency is positively correlated to inspection time (Spearman R = 0.43, *p* = 0.02; N = 29).

### The relationship between egg touching and recognition

**Fig 2:**
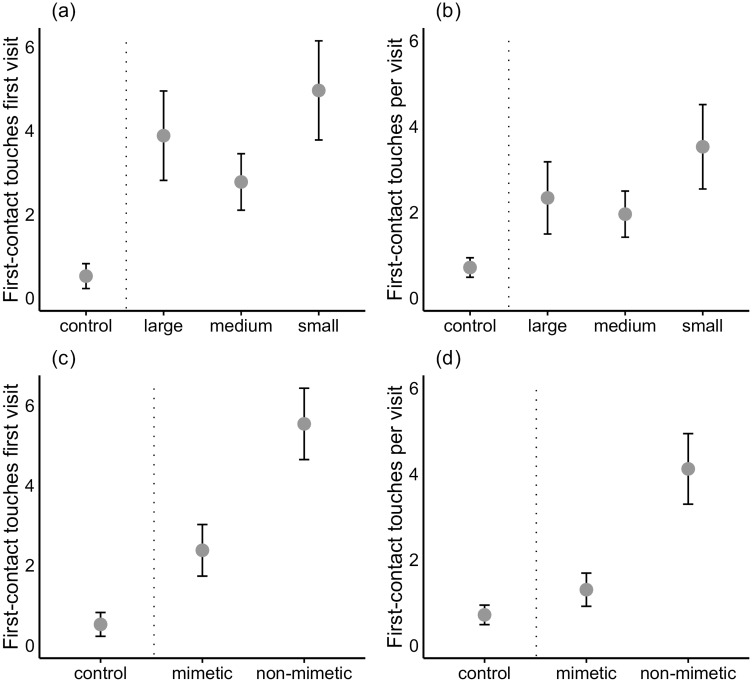
Recognition of the parasitic egg. First-contact touches during the first visit with respect to (a) size mimicry and (c) colour mimicry of the parasitic egg; and first-contact touches per visit with respect to (b) size mimicry and (d) colour mimicry of the parasitic egg. The figure also includes information from control nests (the dashed line separates control nests and those containing experimental eggs).

Blackbird females did not modify their number of first-contact touches during their first visit on account of egg size (χ^2^ = 2.63; *p* = 0.62; Fig 2a). However, colour significantly affected this variable (χ^2^ = 14.6; df = 1; *p* = 0.001) showing that non-mimetic eggs were touched significantly more often (5.54 ± 0.89 touches) than mimetic eggs (2.38 ± 0.65 touches) by females during their first visit (Fig 2c). Interestingly, we found an important effect of clutch size on the number of touches during the first visit (χ^2^ = 12.6; df = 1; *p* = 0.002) showing that females gave significantly more touches in clutches of two (5.89 ± 1.03 touches) than three eggs (2.00 ± 0.39 touches). Furthermore, during the first visit, blackbird females touched non-mimetic eggs more often that eggs of control nests (5.54 ± 0.89 and 0.52 ± 0.3 female touches during the first visit, respectively; z = 4.50, p < 0.001; Fig 2c). The interaction between colour and size did not show a significant effect for female touches during their first visit (χ^2^ = 4.68; df = 1; *p* = 0.32).

In relation to the number of touches per visit (considering the complete filming period), there was again no size-related effect (χ^2^ = 4.47; df = 1; *p* = 0.35; Fig 2b). However, we found a significant effect of egg colour (χ^2^ = 16.46; df = 1; *p* < 0.001) as females touched non-mimetic eggs significantly more often (4.12 ± 0.82 touches/visit) than the mimetic ones (1.30 ± 0.39 touches/visit; Fig 2d). Similarly, as for the first visit, there was no significant interaction between colour and size (χ^2^ = 2.73; df = 1; *p* = 0.60). Interestingly, eggs in nests parasitized with non-mimetic experimental eggs were touched significantly more often (per visit) than those in control nests (4.12 ± 0.82 and 0.71 ± 0.23 touches/visit, respectively; z = 2.47, *p* = 0.01; Fig 2d). We did not find significant differences for this variable between the group with medium-sized mimetic eggs (1.06 ± 0.68 touches/visit) and controls (0.71 ± 0.23 touches/visit; z = -0.82, p = 0.41). Finally, neither colour mimicry, size nor their interaction significantly affected the number of incubation touches (all p-values > 0.11).

Blackbirds touched the eggs less frequently during their last visit in comparison with their first visit (χ^2^ = 16.94; df = 1; *p* < 0.0001). However, this effect was more pronounced with non-mimetic eggs (χ^2^ = 7.24; df = 1; *p* = 0.007) and we did not find any effect of colour on first-contact touches for the last visit (χ^2^ = 3.57; df = 1, *p* = 0.18). Egg size also had an important effect on the frequency of touches between the first and last visit (χ^2^ = 22.31; df = 2; *p* < 0.0001) as blackbirds reduced their number of touches of large (χ^2^ = 8.66; df = 1; *p* = 0.01) and small eggs (χ^2^ = 8.46; df = 1; *p* = 0.01) but not medium-sized eggs (χ^2^ = 1.64; df = 1; *p* = 0.64) during the last visit. The significant three-way interaction between colour, size and visit (χ^2^ = 7.04; df = 2; *p* = 0.029) indicates that our treatment affected the reduction in touches between the first and last visits. Thus, we found no differences in touches between these two visits for both non-mimetic medium-sized eggs (χ^2^ = 0.19; df = 1; *p* = 0.66) and mimetic large eggs (χ^2^ = 4.23; df = 1; *p* = 0.12).

### Relationships between recognition and ejection

Nests fall into four categories according to the relationships between egg recognition (first visit) and egg ejection: those in which (a) the egg was touched (more frequently than in 95% of control nests) and ejected (25.4%), (b) the egg was not touched but was ejected (22.2%), (c) the egg was touched and accepted (17.5%), and (d) the egg was not touched and not accepted (34.9%). The rejection rate was therefore 48% and blackbirds recognized (i.e. touched) the eggs but decided not to eject them in 17.5% of nests. This result contradicts Prediction 3a, but supports the alternative Prediction 3b. The number of first-contact touches per visit varied significantly between these groups of nests (χ^2^ = 47.92; df = 1; *p* < 0.0001) but, more importantly, there is no difference between the number of first contact touches per visit in nests in which the egg is ejected (3.74 ± 0.9 touches/visit) and those in which it is not ejected (2.39 ± 0.73 touches/visit; χ^2^ = 2.04; df = 1; *p* = 0.36). We obtained similar results when considering only the first visits (nests in which the egg is ejected = 5.17 ± 1.07 touches, and those in which it is not ejected = 3.67 ± 0.89 touches (χ^2^ = 1.04; df = 1; *p* = 0.59)).

We also found a nearly significant negative correlation between female touches and ejection latency (First visit: Spearman R = -0.36, *p* = 0.058; per visit: Spearman R = -0.61, p < 0.001; N = 29). Hence, the more the female touched the eggs the sooner they were ejected.

## Discussion

### Immediate ejection and ejection latency

We found that both the degree of mimicry and the size of the parasitic egg have a significant effect on the immediate ejection rate. Thus, non-mimetic model eggs were ejected more frequently at the onset (i. e. within two hours after parasitism). Many studies have documented that the egg rejection latency both within and among host species is highly variable [22, 24, 29, 30]. However, the reasons for such variation are poorly understood. Our first prediction stated that the time taken to eject large parasitic eggs will be longer than for smaller eggs because it is more difficult for the female to pick up a large egg with her beak. Although we were unable to test this prediction since only one large egg was ejected, thus preventing the inclusion of this group in this analysis, we have found that the ejection interval was longer for medium-sized than for small experimental eggs, partly supporting our first prediction.

The effect of egg size on ejection latency in the case of medium-sized eggs could be due to two different reasons: (i) they are harder to recognize because of their better mimicry (in size) than small eggs, or (ii) they are harder to be eject because they are more difficult to pick up. Our results related to egg-touching behaviour suggest that egg size is not an important factor in the recognition of the parasitic egg, so that the delay in ejecting medium-sized eggs is probably because their larger size hinders the act of ejection. These results are important from a theoretical point of view because they imply that difficulty in egg ejection is an important factor that affects ejection latency, which can be related to some physical characteristic of the parasitic eggs that are not necessarily linked to egg recognition. These findings are supported by a recent experimental study that reported that heavier (but same-sized) eggs were ejected less frequently by blackbird females than normal-weight or light eggs [32].

In agreement with our second prediction, we have found that non-mimetic eggs were ejected sooner than mimetic ones. This result accords with previous findings reported for several species [24, 28, 39, 40, 41], which suggests that difficulty in egg recognition is the principal factor affecting ejection latency. However, our results show that difficulty in egg ejection also seems to be an important factor, affecting not only ejection latency (this study), but also the decision to eject [32].

Clutch inspection is another important factor affecting ejection latency given that parasitized hosts need time to process the visual characteristics of the eggs because of recognition problems [24, 30]. Indeed, females of three egg-puncturing ejector species that looked at their parasitized clutches for longer periods ejected the experimental egg sooner than females that inspected them for a shorter time [21, 24, 30]. Surprisingly, we have found the opposite result: ejection latency was positively correlated with time looking at the eggs in the grasp-ejector blackbird. Moreover, we have also found that the more the female touched the eggs the sooner they were ejected. These findings have important theoretical implications for the proximate mechanisms driving egg-rejection behaviour (see below).

### Relationships between egg-touching and egg-recognition

Weak pecking by hosts when confronted with an experimental egg has been reported several times but this behaviour has traditionally been interpreted as trials to puncture the experimental egg [22, 24, 25, 39, 54, 55]. Currently, egg-pecking behaviour is considered evidence of egg recognition even if ejection does not occur [21, 22–25, 32]. In several species egg pecking is a clear behaviour directed only against an experimental (foreign) egg and is thus considered an unambiguous demonstration of egg recognition [21, 25]. This is not the case in the blackbird, perhaps because it is a grasp-ejector species. In the blackbird the experimental egg is not clearly pecked, but touched (some eggs in control nests are also touched). However, two pieces of evidence show that egg touching is a clear indication of egg recognition in this species. First, we have found that blackbird females touched the eggs in experimental nests more frequently than those in control nests. Second, clutches with non-mimetic eggs were touched significantly more often than those with mimetic eggs (Fig 1b in this study, [32]). Evidence that egg-touching (or egg-pecking) implies egg recognition has been reported in a nest-deserter species [23] and in two egg-puncturing species [21, 25]. The blackbird, a grasp-ejector, can now be added to the list of species in which egg touching is considered an indicator of egg recognition ([32], this study).

### Egg recognition without ejection

In nearly 18% of the experimental nests in which the eggs were touched, the experimental eggs were not subsequently ejected. This contradicts our Prediction 3a, but supports the alternative prediction 3b, which confirms that host species, at least sometimes, recognize more eggs than they reject [21,25,31,32]. Recognition without rejection has been experimentally demonstrated in three other species. Repeated parasitism of yellow warblers (*Setophaga petechia*) nests revealed that after recognizing a parasitic egg, individuals may either accept it or desert the nest [31]. Antonov *et al*. [25] showed that eastern olivaceous warblers (*Hippolais pallida*) pecked the experimental egg very often, but only half of such eggs were finally ejected. Finally, female rufous-tailed scrub robins, which were able to eject the experimental egg easily by grasping it, also frequently (55% of cases) pecked the experimental egg but did not eject it [21]. The fact that sometimes mimetic and larger experimental eggs are recognized but accepted implies that motivation is crucial to reaching the threshold needed to decide whether or not to eject, as previously suggested [21, 24, 32, 42].

Egg-recognition studies traditionally assume that rejection implies recognition whereas acceptance implies absence of recognition [2–4]. However, the fact that hosts often recognize the parasitic egg but do not reject it (as demonstrated in the four host species tested so far, including the blackbird) implies that the recognition rate is higher than rejection rate, i.e. that rejection is not always the result of recognition. In fact, our results show that accepting parasitic eggs is not always the consequence of recognition failure. Instead they demonstrate that, at least sometimes, it is the consequence of acceptance decisions, a finding that should be taken into account in future studies on egg recognition.

### Proximate mechanisms

We have found that blackbird females eject non-mimetic eggs sooner than mimetic experimental eggs, which is in agreement with previous findings [21, 24, 25]. This result supports the idea that ejection of mimetic eggs should need a longer time given that they are more difficult to recognize, making recognition errors more possible [42, 56]. However, our finding that blackbird females that inspected their clutches for longer periods of time ejected the parasitic eggs later than females that inspected their clutches for a shorter period of time is not in agreement with previously published results for three different species [21, 24, 30]. Two of these three species are puncture ejectors, and another rejects by nest-desertion, but the cognitive mechanisms involved in the egg rejection process [19, 21, 57] should not be different depending on the rejection method (desertion, puncturing or grasping). An important difference between those three species and the blackbird, that could explain the above mentioned difference, is that the former are current cuckoo hosts with intermediate ejection rates (see Appendix in [2]), while the latter is not currently used as a host by cuckoos and presents a high rejection rate of non-mimetic eggs [35]. The perception of risk of parasitism by hosts increases in the presence of brood parasites, which consequently increases ejection rates [10, 58]. Thus, absence of parasitism implies absence of stimuli related to activity of brood parasites near host nests, which would promote rejection. Such absence would reduce motivation to eject because the threshold needed to decide ejection would be higher. This implies that the motivation to eject is lower in species that are not currently used as hosts and so the decision to eject an experimental egg will involve more time spent inspecting the eggs.

Blackbird females touched the eggs more frequently during their first visit to the nest after the introduction of the experimental egg than during the last filmed visit. Moreover, the difference in the number of touches between these two visits is significantly higher for non-mimetic (either in colour or size) than for mimetic eggs. This means that when an experimental egg is recognized this occurs immediately after the females’ return to the nest. Thus, our results show that the delay in egg ejection is mostly the consequence of a delay in the decision to reject (i.e. the second step in the egg-rejection process). This decision needs an increase in motivation, and so takes longer [21].

The time spent on each of the three stages of the egg-rejection process (recognition, decision and ejection [21]) probably depends on the costs associated with each of them. Where the risk of making recognition errors is high (hosts could reject their own oddly coloured eggs [7, 9, 42, 56, 59]), hosts will inspect their clutches for longer until they are confident that the odd egg is different enough to be considered foreign. This would be the case with a foreign mimetic egg and it has in fact been suggested that the recognition problem is the main factor affecting ejection latency [24, 28, 39–41]. However, ejection costs (hosts could damage their own eggs while trying to eject the parasitic egg [7, 59–61]) are also known to be potentially important in affecting ejection latency because eggshell strength may impede egg-puncturing ejection [62, 63]. Furthermore, the size and weight of the foreign egg are also known to impede ejection in both egg-puncturing and egg-grasping ejector species [24, 32, 38]. Thus, a delayed decision to reject could be due not only to recognition problems as usually assumed (see references above), but also to potential difficulties in ejection. Therefore, the interval between recognition and the act of ejection (decision phase) is devoted to assessing (always in relation to the risk of parasitism) both whether or not the putative foreign egg really is foreign, and the potential costs of egg ejection. In fact, a recent blackbird study has found that heavy eggs previously recognized as foreign were more frequently accepted than light or control eggs, the consequence of motivation not being high enough to enable blackbirds to assume the higher costs that the ejection of a heavy egg could impose [32]. Thus, the key element deciding the fate of the experimental egg throughout the egg rejection process is the motivation to reject together with a flexible rejection threshold based on phenotypic plasticity and risk of parasitism [21].

In conclusion, our results with blackbirds show that (1) egg touching is a reliable indication of recognition even in the absence of egg ejection, (2) the delay in egg ejection does not seem to be due to recognition problems but is mainly the consequence of a delay in the decision to reject, (3) accepting parasitic eggs, at least sometimes, is the consequence of acceptance decisions provoked by the recognition constraints imposed by highly mimetic eggs or by mechanical constraints imposed by larger (i.e. harder to eject) eggs, and (4) given the potential costs associated with the rejection process, motivation is essential to deciding whether or not to eject, needing to be strong enough to reach the threshold needed to trigger the act of ejection. More egg-recognition experiments that film host behaviour when confronted with a foreign egg are needed to fully understand the proximate mechanisms driving the egg recognition and egg rejection processes.

## Supporting information

S1 Table Data used in this paper. (PDF)

S1 Video Blackbird females touching and ejecting an experimental non-mimetic egg. (MP4)
