# Supplementary material for: Relationships between egg-recognition and egg-ejection in a grasp-ejector species
Source: PLoS One. 2017 Feb 7;12(2):e0166283. doi: 10.1371/journal.pone.0166283 (PMC5295713; doi:10.1371/journal.pone.0166283)
Supplement: S1 Table — (PDF) [file pone.0166283.s001.pdf]

N_id	colour	size	date	clutch	finish	eject	i_eject	e_time	inspect	tfv	tlv	tpv	tdi	group
3	M	l	13	two	1	0	0	NA	0 ,073	0	0	0	3 ,24	D
10	M	l	7	three	1	0	0	NA	0 ,061	0	0	0	15 ,077	D
11	NM	l	10	two	1	0	0	NA	0 ,113	2	0	0 ,667	23 ,969	D
13	NM	l	12	two	1	0	0	NA	0 ,142	14	0	3 ,167	12 ,5	C
15	NM	l	3	three	0	NA	0	NA	0 ,02	0	0	0	0	NA
16	NM	s	3	three	1	1	1	0 ,706	0 ,348	0	2	0 ,5	8 ,612	B
20	NM	l	3	three	0	NA	NA	NA	0	NA	NA	NA	NA	NA
21	NM	s	8	two	1	1	0	12	0 ,119	12	0	6	56 ,772	A
23	M	l	12	two	0	NA	0	NA	0 ,066	5	1	3	39 ,146	NA
24	con	con	11	two	NA	NA	NA	NA	NA	0	NA	1,333	20,56	NA
25	M	l	7	three	1	0	0	NA	0 ,05	1	0	0 ,2	0	D
26	M	s	7	two	1	1	0	36	0 ,001	0	0	0	0	B
27	con	con	10	two	NA	NA	NA	NA	NA	5	NA	2,5	13,11	NA
28	con	con	9	two	NA	NA	NA	NA	NA	0	NA	0	16,74	NA
29	con	con	20	three	NA	NA	NA	NA	NA	0	NA	0	48,62	NA
30	M	s	7	two	1	1	NA	12	0	NA	NA	NA	NA	B
33	M	l	16	two	1	0	0	NA	0 ,092	7	8	3 ,857	23 ,052	C
35	NM	l	15	two	1	0	0	NA	0 ,037	1	2	1 ,167	24 ,421	C
39	NM	s	20	two	1	1	1	0 ,289	0 ,007	16	NA	16	0	A
41	NM	s	10	two	1	1	1	8 ,108	0 ,006	5	1	2 ,333	0	A
44	con	con	10	three	NA	NA	NA	NA	NA	0	NA	0	0	NA
45	M	s	10	three	1	1	1	1 ,129	0 ,081	2	1	1 ,333	36 ,323	B
46	M	s	16	two	1	0	0	NA	0 ,121	7	0	1 ,875	14 ,439	C
47	M	l	10	three	1	0	0	NA	0 ,369	10	0	4	88 ,366	C
48	M	s	13	two	1	1	0	36	0 ,111	21	3	9 ,75	52 ,153	A
49	M	s	25	two	1	0	0	NA	0 ,004	2	0	1	0	D
50	NM	s	12	two	1	1	0	12	0 ,022	6	1	3 ,5	24 ,73	A
57	con	con	13	three	NA	NA	NA	NA	NA	0	NA	0	17,47	NA
60	NM	s	14	three	1	1	1	0 ,544	0 ,042	5	NA	5	126 ,788	A
62	M	l	20	two	1	0	0	NA	0 ,066	0	0	0	6 ,698	D
63	con	con	16	three	NA	NA	NA	NA	NA	0	NA	1,273	6,35	NA
65	M	l	36	two	1	0	0	NA	0 ,044	1	0	0 ,5	22 ,568	D
70	NM	s	17	three	1	1	0	12	0 ,095	5	0	1 ,667	57 ,392	A
73	NM	s	33	two	1	1	1	0 ,301	0 ,022	15	NA	15	0	A
75	M	s	18	two	0	NA	0	NA	0 ,075	5	0	1 ,667	12 ,463	NA
76	NM	l	19	two	1	0	0	NA	0 ,102	9	1	5	33 ,504	C
77	con	con	19	three	NA	NA	NA	NA	NA	0	NA	1,5	25,16	NA
78	M	l	19	three	1	0	0	NA	0 ,126	0	0	0	63 ,866	D
80	NM	l	19	three	1	0	0	NA	0 ,098	0	0	0	35 ,673	D
88	NM	l	24	three	0	NA	0	NA	0 ,174	2	0	1	34 ,244	NA
90	NM	s	24	three	1	1	0	12	0 ,169	3	0	0 ,75	46 ,019	B
92	NM	s	25	two	0	NA	0	NA	0 ,212	1	0	0 ,5	44 ,905	NA
93	con	con	26	two	NA	NA	NA	NA	NA	0	NA	0	8,6	NA
94	M	s	38	two	1	0	0	NA	0 ,009	0	0	0	0	D
95	NM	l	26	two	0	NA	0	NA	0 ,001	0	NA	0	0	NA
98	M	s	27	three	0	NA	0	NA	0 ,507	0	4	2	0	NA
104	M	s	27	three	1	1	0	12	0 ,003	0	0	0	0	B
109	NM	l	33	three	1	0	0	NA	0 ,086	4	0	1 ,333	13 ,864	D
111	M	s	28	three	1	0	0	NA	0 ,108	0	0	0	35 ,027	D

112	NM	s	28	two	1	1	1	0 ,077	0 ,006	16	NA	16	0	A
113	NM	l	29	two	1	0	0	NA	0 ,12	20	NA	20	28 ,013	C
114	M	l	30	two	1	0	0	NA	0 ,088	12	6	6 ,667	28 ,608	C
115	M	l	28	three	1	0	0	NA	0 ,07	1	0	1	20 ,124	D
116	M	l	28	three	0	NA	0	NA	0 ,029	0	0	0	0	NA
117	NM	s	28	three	0	NA	0	NA	0 ,057	5	NA	5	34 ,286	NA
121	con	con	29	two	NA	NA	NA	NA	NA	0	NA	0	8,12	NA
125	NM	s	33	three	0	NA	0	NA	0 ,043	1	0	0 ,2	14 ,161	NA
127	M	l	39	two	0	NA	0	NA	0 ,038	1	0	0 ,333	6 ,32	NA
128	NM	l	37	two	1	1	1	0 ,093	0 ,005	6	NA	6	0	A
130	con	con	37	three	NA	NA	NA	NA	NA	0	NA	0	16,13	NA
132	con	con	38	two	NA	NA	NA	NA	NA	0	NA	0	20,01	NA
135	NM	m	44	three	1	0	0	NA	0 ,029	2	5	2 ,333	3 ,832	C
137	M	m	44	three	1	1	0	108	0 ,037	0	0	0	8 ,538	B
138	M	s	38	three	1	1	0	12	0 ,06	1	0	0 ,667	22 ,309	B
139	M	l	39	three	1	0	0	NA	0 ,156	1	0	0 ,5	36 ,346	D
140	M	s	39	three	1	1	0	60	0 ,125	0	0	0	40 ,602	B
141	NM	m	44	two	1	1	0	84	0 ,084	5	0	2	19 ,764	A
147	M	m	44	three	1	0	0	NA	0 ,041	0	0	0	16 ,914	D
149	NM	s	41	three	1	1	1	0 ,068	0 ,002	1	NA	1	0	B
154	con	con	41	three	NA	NA	NA	NA	NA	1	NA	0,333	0	NA
155	NM	m	45	three	1	1	1	1 ,627	0 ,079	7	5	6	64 ,597	A
158	M	m	44	three	0	NA	0	NA	0 ,013	2	0	1	0	NA
160	M	m	46	three	0	NA	0	NA	0 ,002	0	0	0	0	NA
162	NM	m	44	three	1	1	1	0 ,351	0 ,042	3	NA	3	203 ,094	B
171	NM	m	45	three	1	0	0	NA	0 ,012	5	NA	5	0	C
173	M	m	46	three	0	NA	0	NA	0 ,003	0	NA	0	2 ,637	NA
175	con	con	45	two	NA	NA	NA	NA	NA	0	NA	0	9,39	NA
178	NM	m	51	two	0	NA	NA	NA	0	NA	NA	NA	NA	NA
183	M	m	48	two	1	1	0	36	0 ,095	0	0	1	18 ,969	B
186	NM	m	53	two	0	NA	0	NA	0 ,095	14	1	7 ,5	15 ,635	NA
187	NM	m	49	three	1	1	0	84	0 ,033	8	0	0 ,333	4 ,636	B
189	M	m	51	two	1	1	0	84	0 ,044	0	0	0	21 ,384	B
194	NM	m	54	two	1	1	0	108	0 ,121	0	0	0 ,333	13 ,671	B
196	NM	m	53	two	1	0	0	NA	0 ,289	1	18	4 ,4	30 ,527	C
197	M	m	50	three	0	NA	0	NA	0 ,048	4	0	0 ,8	25 ,245	NA
200	con	con	49	three	NA	NA	NA	NA	NA	0	NA	1,25	15,93	NA
208	M	m	53	two	1	0	0	NA	0 ,176	1	0	0 ,333	6 ,542	D
209	M	m	58	two	1	0	0	NA	0 ,045	13	NA	13	41 ,945	C
212	con	con	72	three	NA	NA	NA	NA	NA	0	NA	0	99,86	NA
214	M	m	54	two	0	NA	0	NA	0 ,63	0	0	0	40 ,084	NA
220	NM	m	58	two	1	0	0	NA	0 ,021	2	0	0 ,667	37 ,163	D
221	M	m	66	three	1	0	0	NA	0 ,065	5	0	2 ,333	39 ,211	C
225	M	m	60	three	0	NA	0	NA	0 ,159	0	0	0	34 ,951	NA
231	M	m	63	three	1	0	0	NA	0 ,043	0	0	0	12 ,779	D
236	NM	m	66	three	1	1	1	0 ,1	0 ,001	2	NA	2	0	B
237	con	con	69	two	NA	NA	NA	NA	NA	4	NA	3,25	20,16	NA
239	NM	m	68	three	1	1	1	0 ,227	0 ,006	7	NA	7	0	B
240	con	con	51	three	NA	NA	NA	NA	NA	1	NA	0,5	0	NA
241	M	m	69	three	1	0	0	NA	0 ,223	0	NA	0	6 ,222	D

242	con	con	51	three	NA	NA	NA	NA	NA	0	NA	2,8	38,6	NA
243	con	con	73	three	NA	NA	NA	NA	NA	0	NA	0,2	52,58	NA
39X	M	m	65	three	1	0	0	NA	0 ,08	0	0	0	25 ,801	D
43X	M	m	65	two	1	1	0	84	0 ,096	4	0	1 ,333	35 ,953	B
50X	M	m	61	three	0	NA	0	NA	0 ,143	1	1	0 ,286	41 ,845	NA
85X	M	m	53	three	0	NA	0	NA	0 ,244	0	0	0	41 ,296	NA
11X	con	con	48	three	NA	NA	NA	NA	NA	0	NA	0	15,21	NA
